# The cost of mapping trachoma: Data from the Global Trachoma Mapping Project

**DOI:** 10.1371/journal.pntd.0006023

**Published:** 2017-10-18

**Authors:** Guillaume Trotignon, Ellen Jones, Thomas Engels, Elena Schmidt, Deborah A. McFarland, Colin K. Macleod, Khaled Amer, Amadou A. Bio, Ana Bakhtiari, Sarah Bovill, Amy H. Doherty, Asad Aslam Khan, Mariamo Mbofana, Siobhain McCullagh, Tom Millar, Consity Mwale, Lisa A. Rotondo, Angela Weaver, Rebecca Willis, Anthony W. Solomon

**Affiliations:** 1 Research Department, Sightsavers, Haywards Heath, United Kingdom; 2 Neglected Tropical Diseases Department, Sightsavers, Haywards Heath, United Kingdom; 3 Rollins School of Public Health, Emory University, Atlanta, GA, United States of America; 4 Clinical Research Department, London School of Hygiene & Tropical Medicine, London, United Kingdom; 5 Prevention of Blindness Programme, Ministry of Health, Cairo, Egypt; 6 Programme National de Lutte Contre les Maladies Transmissibles, Ministère de la Santé, Cotonou, Benin; 7 Task Force for Global Health, Atlanta, GA, United States of America; 8 RTI International, Washington, D.C., United States of America; 9 King Edward Medical University, Lahore, Pakistan; 10 Health Programa Nacional de Oftalmologia, Ministerio da Saude, Maputo, Moçambique; 11 Provincial Health Office, Ndola, Zambia; 12 United States Agency for International Development, Washington, D.C., United States of America; 13 Department of Control of Neglected Tropical Diseases, World Health Organization, Geneva, Switzerland; College of Health Sciences, Bayero University Kano, NIGERIA

## Abstract

**Background:**

The Global Trachoma Mapping Project (GTMP) was implemented with the aim of completing the baseline map of trachoma globally. Over 2.6 million people were examined in 1,546 districts across 29 countries between December 2012 and January 2016. The aim of the analysis was to estimate the unit cost and to identify the key cost drivers of trachoma prevalence surveys conducted as part of GTMP.

**Methodology and principal findings:**

In-country and global support costs were obtained using GTMP financial records. In-country expenditure was analysed for 1,164 districts across 17 countries. The mean survey cost was $13,113 per district [median: $11,675; IQR = $8,365-$14,618], $17,566 per evaluation unit [median: $15,839; IQR = $10,773-$19,915], $692 per cluster [median: $625; IQR = $452-$847] and $6.0 per person screened [median: $4.9; IQR = $3.7-$7.9]. Survey unit costs varied substantially across settings, and were driven by parameters such as geographic location, demographic characteristics, seasonal effects, and local operational constraints. Analysis by activities showed that fieldwork constituted the largest share of in-country survey costs (74%), followed by training of survey teams (11%). The main drivers of in-country survey costs were personnel (49%) and transportation (44%). Global support expenditure for all surveyed districts amounted to $5.1m, which included grant management, epidemiological support, and data stewardship.

**Conclusion:**

This study provides the most extensive analysis of the cost of conducting trachoma prevalence surveys to date. The findings can aid planning and budgeting for future trachoma surveys required to measure the impact of trachoma elimination activities. Furthermore, the results of this study can also be used as a cost basis for other disease mapping programmes, where disease or context-specific survey cost data are not available.

## Author summary

There are currently few data sets available to aid programmes in planning and budgeting for population-based surveys in low- and middle- income countries. With the objective of identifying cost drivers and key variables influencing prevalence survey costs, the authors collected expenses incurred during the Global Trachoma Mapping Project (GTMP) which surveyed 2.6 million people across 29 countries. Expenditure from surveying 1,164 districts in 17 countries was analysed. Our results showed that the majority of in-country expenditure was spent on personnel (per diems, accommodation, meals and beverages) (49%) and local transportation (44%) and that the median survey expenditure was US$11,675 per district (or US$15,839 per evaluation unit, US$625 per cluster and US$4.9 per person examined). There were large variations in survey unit costs across settings, based on local geographic, demographic, seasonal effects and local operational characteristics. In addition, the resources required for the global support and coordination of the GTMP were analysed and amounted to US$5.1m (US$3,318 per district or US$5,668 per EU). Global support expenses can be substantial for a large multi-country mapping exercise conducted in a limited period of time such as the GTMP. Findings from this study can be used to inform other disease mapping projects, and to inform planning and budgeting for the prevalence surveys that will assess the impact of trachoma elimination interventions.

## Introduction

An estimated 285 million people worldwide live with visual impairment, including 39 million who are blind [1]. Trachoma is the leading infectious cause of avoidable blindness, responsible for the visual impairment of about 1.9 million people, of whom 0.5 million are irreversibly blind [2]. The disease is a public health problem in 42 countries, with just over 200 million people being at risk, the majority of whom are in Sub-Saharan Africa [3].

The World Health Organization (WHO) has called for global elimination of trachoma as a public health problem by 2020. To achieve it, WHO recommends the use of a strategy known as SAFE. This includes (i) Surgery to correct the advanced stage of the disease, known as trachomatous trichiasis (TT), when one or more eyelashes rub on the eyeball; (ii) Antibiotics to clear ocular *Chlamydia trachomatis* infection, and (iii) Facial cleanliness and (iv) Environmental improvement to reduce infection transmission. The “S” component targets individuals, while “A”, “F” and “E” are community-based interventions. WHO recommends that “A”, “F” and “E” are delivered for at least three years in districts in which ≥10% of children aged 1–9 years are estimated to have the sign trachomatous inflammation-follicular (TF), increasing to 5 years in districts in which ≥30% of children have TF. Data on the prevalence of TF and TT at district level (where “districts” generally contain populations of 100,000–250,000 people) are therefore vital for planning interventions and judging the subsequent success of those efforts [4].

**Fig 1:**
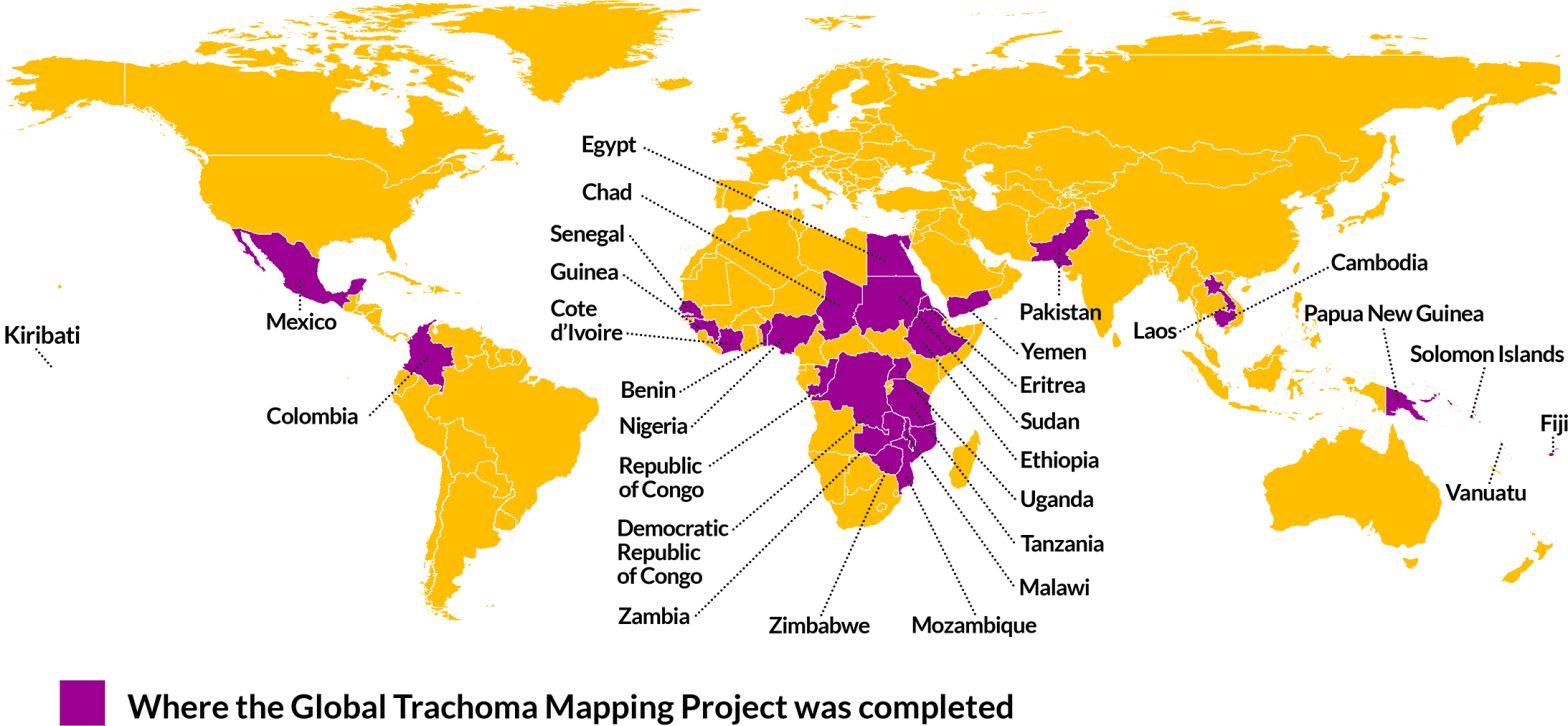
Countries Surveyed as Part of the Global Trachoma Mapping Project. Fig 1 was adapted from an open source map retrieved on Natural Earth Data at http://www.naturalearthdata.com/about/terms-of-use.

The Global Trachoma Mapping Project (GTMP) was launched in 2012, with the aim of completing the baseline map of trachoma worldwide by estimating the prevalence of TF and TT in all suspected trachoma-endemic areas for which prevalence data were unavailable. Between December 2012 and January 2016 the GTMP examined more than 2.6 million people across 29 countries (Fig 1).

In addition to providing the evidence base for commencing district-wide implementation of the SAFE strategy, prevalence surveys are also used to evaluate the impact of trachoma elimination interventions. The need for such surveys will increase in the near future, as elimination efforts are ramped up globally. The cost of population-based surveys has been cited as a reason for delays in conducting impact and surveillance surveys, so providing data on actual costs is important to inform debate [5]. The existing literature on the economics of trachoma elimination is sparse, with only one published study addressing the incremental costs of prevalence surveys [5], and several other papers focusing on socioeconomic burden, and the cost-effectiveness of interventions [6–9].

## Methods

Surveys were implemented by health ministries, supported by local nongovernmental organisation (NGO) partners. All surveys followed a standardised methodology, as described in detail elsewhere [4].

### Survey design

Surveys were carried out in areas defined as evaluation units (EUs). Evaluation units were used to address two issues: (1) local definitions of a “district” vary greatly in terms of population size and geographical area, and (2) WHO encourages baseline trachoma prevalence surveys to be conducted at larger-than-district level, where trachoma is expected to be highly and widely endemic [10]. EUs (rather than districts) have been used as the principal unit of analysis in order to allow comparability of data on survey costs in this paper. However, cost per districts and clusters are also reported in the tables.

Each EU-level survey followed a multi-stage cluster random sampling methodology. First-level clusters were broadly synonymous with “villages”, or represented the lowest administrative unit covered by census data. In each first-level cluster, teams randomly selected a number of households for inclusion, corresponding to the number of households that a team could screen in one day. To estimate a 10% prevalence of TF with absolute precision of ±3% at the 95% confidence level, 1,019 children aged 1–9 years needed to be sampled, including a design effect of 2.65 to account for the clustered design; this was inflated by a factor of 1.2 to allow for non-response. The number of first-level clusters required varied based on the local demographical situation, with 20 set as a minimum.

Field teams comprised one trachoma grader and one data recorder, and in most cases a driver and a local guide. (In a small number of subprojects, due to local norms, one male grader plus one female grader were needed per team to grade male and female subjects, respectively.) Graders and recorders were recruited by the local health ministry and trained by GTMP-certified trainers, using a standardised training protocol and materials, over five days, consisting of intensive classroom sessions, field based training and testing [4]. In the field, the teams were supervised by an ophthalmologist or senior ophthalmic nurse, who was appointed for every seven to ten teams on average. In selected households, all residents over the age of one year were invited to participate. Consenting individuals were examined for the signs of TT, TF and trachomatous inflammation-intense (TI). Additional questions were asked about households’ access to water and sanitation (WASH), and observation was undertaken of latrine facilities, if present. In some surveys, data were also collected on other diseases of local importance.

All data were captured electronically, using a purpose-built Open Data Kit-based Android smartphone application, and uploaded to a cloud-based server for quality assurance checks (including Global Positioning System (GPS) localisation of households surveyed) and subsequent data cleaning and analysis.

### Costing approach and inclusion criteria

The GTMP was implemented as a series of subprojects. A subproject was a group of surveys conducted in a defined geographical area, for which a unique budget was established and expenditure reports prepared. To ensure comparability across countries and surveys, a number of inclusion criteria were introduced for this study. Mapping subprojects were only included in the costing analysis if they 1) followed the standardised GTMP methodologies; 2) only collected data on trachoma and WASH, or were integrated surveys of trachoma and other diseases in which the trachoma- and WASH-data-related expenditure could be explicitly identified.

In this study, the cost of conducting baseline trachoma surveys included only financial expenditure covered by the GTMP. An activity-based costing accounting model was used to analyse survey expenditure. Data on in-kind contributions and expenditure from project partners or health ministries that would have been incurred in the absence of the mapping project were not collected, with the exception of vehicles. For example, any per diems received by health ministry staff during field work were included, but their base salaries were not. When vehicle time was donated by the health ministry or its partners, a cost estimate was included, based on the duration of mapping and the average daily cost of vehicle hire in previous programmes (mean cost of daily vehicle hire: Malawi MKW90,000, Nigeria NGN15,000, United Republic of Tanzania TZS150,000, Zambia ZMW341, Zimbabwe USD$180).

Finally, administrative costs charged by implementing partners were excluded from the expenditure analysis on the basis that rates were agreed contractually and did not necessarily reflect the actual overhead costs of implementing partners.

### Data collection

All data were collected retrospectively between June 2015 and March 2016. Financial data were obtained via country-specific budgets and expenditure claim forms submitted by in-country implementing partners. Financial data were extracted from the GTMP financial reporting system (CLAIMS) or collected via Excel spreadsheets. Other data, such as the list of EUs, districts, and clusters mapped for each subproject, were provided by the GTMP data managers who cleaned and analysed all field data. Specific information on in-country surveys was also collected through narrative reports and email or phone queries to implementing partners.

### Data analysis

GTMP expenditure was allocated to two categories of activity (in-country survey and global support expenditures) and further divided into five sub-categories as indicated in Table 1.

**Table 1:** Composition of GTMP expenditure.

Overall expenditure				
In-country survey expenditure		Global support expenditure		
Survey implementation	Training	Grant management	Epidemiological support	Data stewardship and processing

Global support expenditure was broken down into three activities: grant management, epidemiological support, and data stewardship and processing.

In-country survey expenditure was allocated to either training or survey implementation sub-categories. Since it was not possible to link training expenditure to specific subprojects (because training activities often involved groups of trainees destined to work in different subprojects), this expenditure has been allocated to each subproject in proportion to the number of EUs surveyed. Details of items included or excluded for each activity and sub-activity are provided in Table 2.

**Table 2:** GTMP in-country survey expenditure composition by activity.

Activity	Definition
***Coordination & planning***	• Included: (i) Personnel and transport expenditure for survey coordination; (ii) Expenditure related to community mobilisation and advocacy events, including sensitisation meetings and mass media; (iii) Costs of contracting a specific organization to co-ordinate partners in Ethiopia, Nigeria and Pakistan, due to the scale of the work and the large number of implementing partners in these countries*. • Excluded: (i) In-country costs of dissemination of survey results (because this activity was not standard and implemented in every country)
***Fieldwork***	• Included: (i) Grader, recorder and village guide per diems, accommodation and transportation expenditure for fieldwork; ii) Supplies (e.g., antibiotics to treat cases of active trachoma identified); (iii) Other expenditures directly attributable to fieldwork activities (e.g., examination loupes, mobile phone data plans to transmit survey data, community based communication aids such as bags, t-shirts or caps, postage). • Excluded: Salaries of health ministry staff
***Supervision***	• Included: (i) Supervisors’ per diems, accommodation, transportation and other expenditure for supervising survey teams during fieldwork. • Excluded: None
***Training***	• Included: (i) Participants’ per diems, accommodation, and transportation expenditure; (ii) Training venue rental; (iii) Supplies (if directly attributable to training activities) • Excluded: (i) International flights and fees for international trainers, epidemiologist salaries—these costs are included in global support costs

*Amounting to US$ 44,889 for Ethiopia, US$ 46,941for Nigeria and US$ 45,956 for Pakistan), these coordination fees were incurred in large countries where a specific organization was tasked with coordinating the work of numerous partners.

Data were collected and analyzed in Excel and Stata 12.0 (Stata Corp, TX, USA). Descriptive statistics were calculated including means, medians and interquartile ranges (IQRs) since in-country survey expenditure by country or subproject did not follow a normal distribution. The IQR includes all values between the 25^th^ and 75^th^ percentile, while outliers are defined as any values above the 95^th^ percentile. It was not possible to allocate survey expenditure to specific EUs, districts or clusters, given that financial data were aggregated and reported by partners at subproject level. The mean unit cost of mapping was hence obtained by dividing the total expenditure by the number of districts, EUs and clusters surveyed in each subproject.

### Currency and purchasing power parity

In-country survey and global operational expenditure were converted in US Dollars (USD) using the mean annual market exchange rates over the mapping period [11]. All USD expenses were then inflated by applying the United States Consumer Price Index, using 2015 as the base year [12]. In order to facilitate cross-country comparisons, the mean survey expenditure per cluster was also converted to International Dollars. An international dollar is a hypothetical currency adjusted by purchasing power parity conversion factors to compensate for price level differences between countries [13]. Therefore, each country’s mean expenditure has been divided by the latest Purchasing Power Parity conversion factors available for each country (PPP, private consumption) [14]. Figures in International Dollars (PPP) are provided in the results section.

### Ethics statement

Ethics clearance for the costing study was granted by the London School of Hygiene & Tropical Medicine ethics committee (reference 11195). The research used routine financial data from the project and did not require additional primary data collection. The underlying epidemiological data were collected during the GTMP and ethics clearance was obtained from the London School of Hygiene & Tropical Medicine (references 6319 and 8355) and from relevant ethical review boards identified by the ministry of health in each country [4]. Informed verbal consent was sought for each survey participant or from the parent or legal guardian when the participant was minor. Verbal consent was preferred over written consent considering that surveys were generally conducted in rural areas with high illiteracy rates. Verbal consent was requested and recorded using the LINKS application (purpose-built application on Android smartphones used for data collection).

## Results

**Fig 2:**
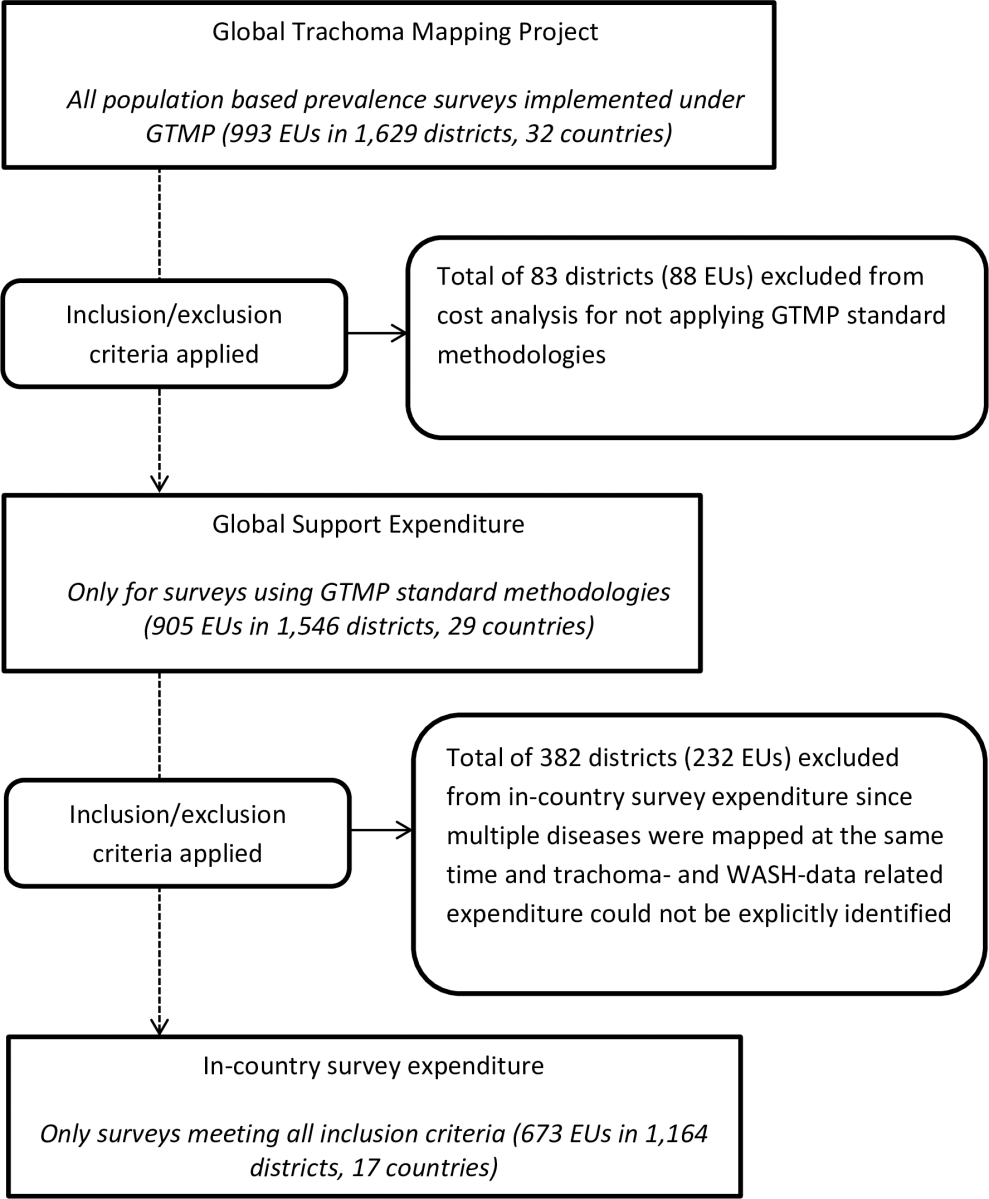
Study inclusion flow diagram.

Baseline trachoma mapping was completed for a total of 1,629 districts (993 EUs) in 32 countries [15–40]. Global support expenditures cover only districts which were mapped using standardised GTMP methodologies: 1,546 districts (905 EUs) in 29 countries. In-country survey expenditures were calculated for 17 countries, including 1,164 districts (673 EUs) which met our study inclusion criteria (Fig 2).

### Global support expenditure

The total cost of the global GTMP support package was $5,129,348 (Table 3). This covered centralised support functions for rolling out the GTMP programme in 29 countries, as well as discussions with a further 20 countries in which mapping, ultimately, was not taken forward for one of a variety of reasons, including excessive security risks or insufficient evidence to justify conducting formal population-based prevalence surveys. Global support expenditure included grant management (40%), epidemiological support (31%), and data stewardship and processing (29%).

**Table 3:** Global GTMP support costs.

Category	Description	Total cost in USD 2015 (%)	Average cost per district in USD 2015 (n = 1,546)	Average cost per EU in USD 2015 (n = 905)
Grant Management*	**Total**	**2,052,597 (40)**	**1,328**	**2,268**
	Audit, bank fees, communications, financial system set up, flights and project management salaries	1,767,438		
	Monitoring and Evaluation	285,159		
Epidemiological support**	**Total**	**1,601,903 (31)**	**1,036**	**1,770**
	Epidemiological Personnel Costs	1,359,015		
	International Flights	242,888		
**Data Stewardship and Processing**	**Total**	**1,474,848 (29)**	**954**	**1,630**
	Data Management	1,022,583		
	Smartphone data collection system Development and Maintenance	261,534		
	Smartphone purchase and shipping	190,731		
**Total Global GTMP Support Cost**		**5,129,348 (100)**	**3,318**	**5,668**

* Includes activities such as international coordination

** Transport and personal cost related to international technical experts who supported ministries of health designing their sampling methodology, plan fieldwork activities; and implement survey.

### In-country survey expenditures

Overall, 89% of in-country survey expenditure was spent on survey implementation and 11% on training. The mean training expenditure per mapping team was $2,357, ranging from $792 per team in Egypt to $8,513 in Pakistan. Survey implementation expenditures included fieldwork (74%), coordination & planning (9%), and supervision (6%). When breaking down in-country survey expenditure by inputs, personnel represented the highest share, accounting for 49% of total spend, followed by transportation (44%), supplies (4%), and other expenditure (3%) (Table 4).

**Table 4:** Percentage of in-country total survey expenditure by input and activity, in 2015 USD.

Inputs	Activity				
		Survey implementation			
	Training (%)	Fieldwork (%)	Coordination & planning (%)	Supervision (%)	Total
Personnel	716,066	2.996,569	680,581	463,892	**4,857,109 (49.5%)**
	(7.3%)	(30.6%)	(6.9%)	(4.7%)	
Transportation	292,633	3,768,103	70,224	143,636	**4,274,596 (43.6%)**
	(3%)	(38.4%)	(0.7%)	(1.5%)	
Supplies	30,151	353,317	44	14	**383,526 (3.9%)**
	(0.3%)	(3.6%)	(0%)	(0%)	
Other*	41,427	113,349	143,606	159	**298,540 (3%)**
	(0.4%)	(1.1%)	(1.5%)	(0%)	
**Total**	**1,080,276**	**7,231,338**	**894,455**	**607,701**	**9,813,770 (100%)**
	**(11%)**	**(73.7%)**	**(9.1%)**	**(6.2%)**	

*Communications expenses (sim cards, mobile data etc.), Insurance, Venue rental and Bank charges

**Fig 3:**
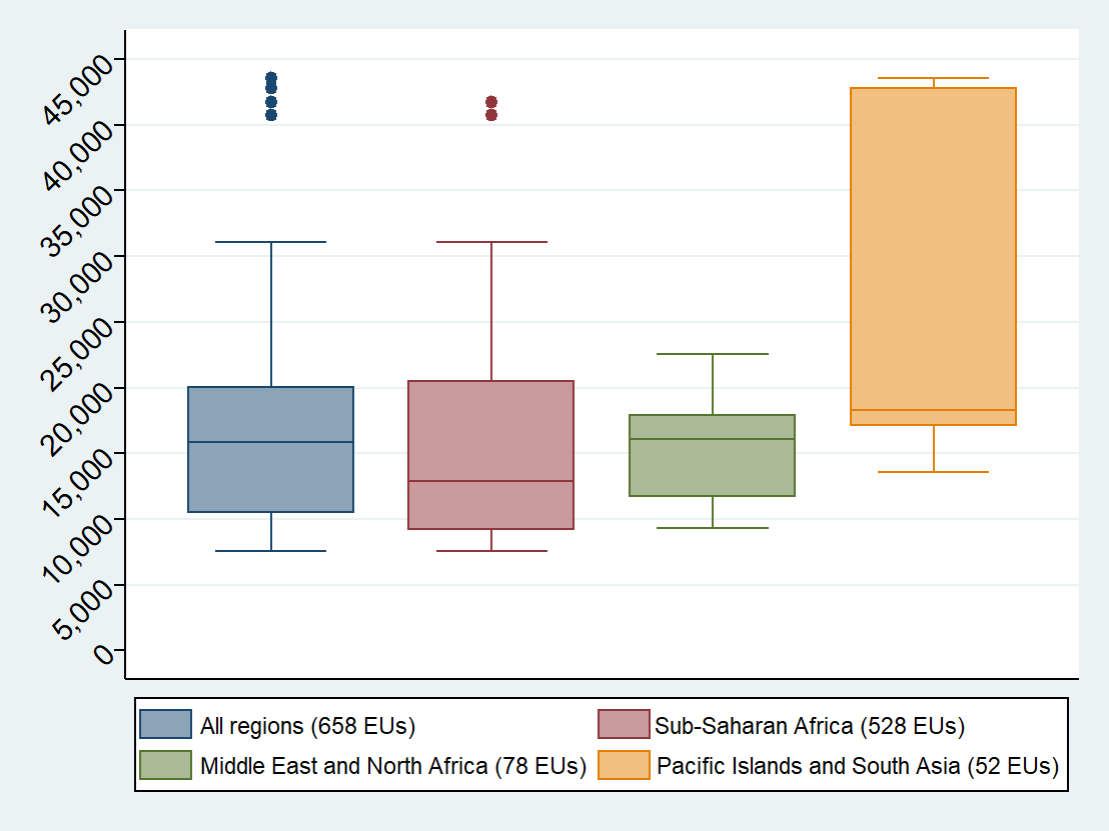
In-country survey expenditure presented by world region (expenditure per EU, in 2015 USD). Vertical boxes show the 25^th^ and 75^th^ percentile of the mean expenditure per EU. The median values are represented by the horizontal bar in each box. Upper and lower bars show extreme values and dots indicate outliers (outliers, defined with Tukey’s method, are values above 3rd quartile plus IQR times 1.5 and values below 1^st^ quartile plus IQR times 1.5 [41]).

The median in-country survey cost was $15,839 per EU, ranging from $7,550 in Niger State, Nigeria to $43,580 in Papua New Guinea. The mean cost per EU was $17,566 (IQR $10,773–$19,915; SD = $9,470). As shown in Fig 3 on the “All regions” box plot, the mean unit cost was higher than the median value because of four outliers: Papua New Guinea ($43,580), Vanuatu ($42,795) and two regions in Ethiopia (Ethiopia Somali region $41,719; Gambella region, $40,767). Fig 3 also shows that subprojects in the Middle East and North Africa had the smallest variance in expenditures per EU, as demonstrated by their relatively low standard deviation (US$ 4,309) compared to the Sub-Saharan African countries (US$ 9,277) and the Pacific Islands and South Asia (US$ 12,777).

At cluster level, the median survey cost was $625, ranging from $290 in Zanzibar, United Republic of Tanzania, to $1,687 in Papua New Guinea (mean of unit costs $692; IQR $452–$847; SD = $350). Outlier values were Papua New Guinea ($1,687), Ethiopia Gambella region ($1,631) and Somali region ($1,558).

A summary of in-country survey expenditure by country and subproject is presented in Table 5. More detailed information is also available in S1 Dataset.

**Table 5:** Summary of in-country survey expenditure.

Country (n = 17)	Mapping subprojects (n = 52)	Number of Districts	Number of EUs	Number of Clusters	In-country Survey Expenditure (total) in USD 2015	Survey Expenditure per District in USD 2015	Survey Expenditure per EU in USD 2015	Survey Expenditure per Cluster in USD 2015	Survey Expenditure per person examined in USD 2015	Survey Expenditure Per Cluster in International $ (PPP, 2014)*,**
**Benin**	**Benin–Alibori, Atacora, Borgou, Donga**	**25**	**10**	**271**	**203,332**	**8,133**	**20,333**	**750**	**4.5**	**1,626**
**Chad**	**All subprojects total**	41	40	972	**456,811**	**11,142**	**11,420**	**470**	**4.1**	**916**
	Chad—Batha, Mayo-kebbi	9	9	221	103,392	11,488	11,488	468	3.8	833
	Chad—Logone Occidental, Tandjile, Borkou Ennedi Tibesti, Guera, Logone Oriental, Moyen Chari, Ndjamena,	17	16	421	206,219	12,131	12,889	490	4.4	953
	Chad—Bahr El Gazel, Chari-baguirmi, Hadjer Lamis, Kanem, Mandoul, Wadi Fira	15	15	330	147,200	9,813	9,813	446	3.9	925
**Egypt**	**Egypt–Lower Egypt**	**4**	**4**	**101**	**60,023**	**15,006**	**15,006**	**594**	**5.8**	**1,194**
**Ethiopia**	**All subprojects total**	**506**	**174**	**4,529**	**3,352,937**	**6,626**	**19,270**	**740**	**6.7**	**1,948**
	Ethiopia—Beneshangul Gumuz	20	7	194	201,755	10,088	28,822	1,040	10.3	2,725
	Ethiopia–Gambella	13	3	75	122,302	9,408	40,767	1,631	15.3	4,352
	Ethiopia—Oromia Total	252	79	2,037	946,407	3,756	11,980	465	4.2	1,206
	Ethiopia—Southern Nations, Nationalities, and Peoples' Region (SNNPR) Total	104	41	1,069	796,551	7,659	19,428	745	6.6	1,945
	Ethiopia—Ethiopia Somali Total	48	18	482	750,937	15,645	41,719	1,558	12.1	4,178
	Ethiopia—Tigray Total	41	15	383	192,745	4,701	12,850	503	5.1	1,309
	Ethiopia- Afar Total	28	11	289	342,240	12,223	31,113	1,184	12.0	3,172
**Ivory Coast**	**All subprojects total**	**11**	**10**	**257**	**163,336**	**14,849**	**16,334**	**636**	**4.2**	**1,578**
	Ivory Coast—Zanzan, Denguele, Bas-Sassandra	4	3	77	54,039	13,510	18,013	702	4.7	1,743
	Ivory Coast—Vallee du Bandama, Worodougou, Montagnes, Savanes	7	7	180	109,298	15,614	15,614	607	4.0	1,508
Malawi***	**All subprojects total**	**25**	**24**	**708**	**482,307**	**19,292**	**20,096**	**681**	**6.4**	**2,041**
	Malawi—Central, Southern	16	16	480	329,449	20,591	20,0591	686	6.8	2,014
	Malawi—Dedza, Mulanje	2	2	48	34,204	17.102	17,102	713	5.8	2,605
	Malawi–Northern	7	6	180	118,654	16,951	19,776	650	5.8	1,965
**Mozambique**	**All subprojects total**	**42**	**29**	**693**	**677,715**	**16,136**	**23,369**	**978**	**9.1**	**1,884**
	Mozambique–Nampula	16	11	264	214,070	13,379	19,461	811	7.5	1,548
	Mozambique–Sofala	12	8	189	201,034	16,753	25,129	1,064	9.4	2,057
	Mozambique—Tete province	14	10	240	262,610	18,758	26,261	1,094	10.7	2,116
Nigeria***	**All subprojects total**	**220**	**220**	**5,546**	**1,886,912**	**8,577**	**8,577**	**340**	**2.4**	**522**
	Nigeria–Bauchi	20	20	500	167,338	8,367	8,367	335	2.3	511
	Nigeria—Benue Total	23	23	575	184,697	8,030	8,030	321	2.0	498
	Nigeria–Federal Capital Territory (FCT)	6	6	150	67,453	11,242	11,242	450	3.9	692
	Nigeria–Gombe	11	11	274	99,374	9,034	9,034	363	2.5	555
	Nigeria–Jigawa	4	4	100	49,158	12,290	12,290	492	5.0	757
	Nigeria–Kaduna	23	23	585	183,718	7,988	7,988	314	2.3	479
	Nigeria–Kano	44	44	1,114	336,183	7,641	7,641	302	2.0	460
	Nigeria–Katsina	34	34	850	308,307	9,068	9,068	363	2.4	555
	Nigeria–Kogi	4	4	125	57,956	14,489	14,489	464	5.2	728
	Nigeria–Kwara	8	8	200	66,868	8,359	8,359	334	2.4	519
	Nigeria–Niger	25	25	625	188,739	7,550	7,550	302	2.5	460
	Nigeria—Kebbi and Sokoto	5	5	124	56,159	11,232	11,232	453	4.2	708
	Nigeria–Taraba	13	13	324	120,960	9,305	9,305	373	2.8	572
**Pakistan**	**All subprojects total**	**49**	**42**	**1,118**	**668,533**	**13,644**	**15,917**	**598**	**4.3**	**1,497**
	Pakistan—Khyber Pakhtunkhwa and Gilgit-Baltistan	14	13	346	222,434	15,888	17,110	643	5.0	1,646
	Pakistan—Azad Jammu and Kashmir	6	4	103	78,346	13,058	19,586	761	4.5	2,032
	Pakistan—Baluchistan and Sindh	10	6	156	109,665	10,967	18,278	703	4.9	1,842
	Pakistan—Punjab	19	19	513	258,088	13,584	13,584	503	3.6	1,184
**Papua New Guinea**	**Papua New Guinea–Madang, National Capital District, Southern Highlands, West New Britain, western Fly**	**6**	**6**	**155**	**261,477**	**43,580**	**43,580**	**1,687**	**16.7**	**1,977**
**Solomon Islands**	**Solomon Islands–Choiseul, Renbel, Temotu, Western**	**4**	**3**	**82**	**53,506**	**13,377**	**17,835**	**653**	**5.8**	**595**
**Sudan**	**All subprojects total**	**45**	**32**	**676**	**535,885**	**11,909**	**16,746**	**793**	**7.3**	**1,397**
	Sudan–Khartoum	6	5	130	46,614	7,769	9,323	359	3.1	663
	Sudan—Central Darfur	7	5	100	83,028	11,861	16,606	830	8.0	1,380
	Sudan—East Darfur	7	4	80	71,645	10,235	17,911	896	8.4	1,488
	Sudan—North Darfur	9	6	120	109,327	12,147	18,221	911	8.5	1,699
	Sudan—South Darfur	8	7	138	112,451	14,056	16,064	815	6.8	1,519
	Sudan—West Darfur	8	5	108	112,820	14,102	22,564	1,045	11.2	1,736
United Republic of Tanzania***	**Zanzibar—Kaskazini Unguja/Pemba**	**2**	**2**	**52**	**15,102**	**7,551**	**7,551**	**290**	**2.3**	**802**
**Vanuatu**	**Vanuatu–Malampa, Penama, Sanma, Shefa, Tafea, Torba**	**1**	**1**	**42**	**42,795**	**42,795**	**42,795**	**1,019**	**11.7**	**866**
**Yemen**	**All subprojects total**	**164**	**42**	**968**	**463,550**	**2,827**	**11,037**	**479**	**3.8**	**1,265**
	Yemen: Al Hudaydah, Al Jawf, Ibb and Ma'rib	70	15	320	148,526	2,122	9,902	464	4.0	1,226
	Yemen: Adh Dhale'a, Hadramoot, Hajjah, Lahj and Taiz	94	27	648	315,023	3,351	11,668	486	3.7	1,285
Zambia***	**Zambia–Central, Copperbelt, Eastern, Luapula, Lusaka, Muchinga, North Western, Northern, Southern, Western**	**3**	**3**	**71**	**33,191**	**11,064**	**11,064**	**467**	**4.2**	**1,390**
Zimbabwe***	**All subprojects total**	**16**	**16**	**385**	**456,358**	**28,522**	**28,522**	**1,185**	**8.6**	**2,280**
	Zimbabwe: Chipinge, Mudzi, Mbire, Kariba, Gokew North, Binga, Mangwe, Chivi	8	8	192	245,250	30,656	30,656	1,277	9.3	2,457
	Zimbabwe: Lupane, Gokwe South, Rushinga, Mutoko, Hurungwe, Hwange, Muzarabani, Uzumba-Maramba-Pfungwe	8	8	193	211,108	26,389	26,389	1,094	7.8	2,104
**Total**		**1,164**	**658**	**16,626**	**9,813,770**					

* Except for Yemen, where 2013 PPP factors were used (latest data available)

** PPP price level ratio factors (instead of PPP conversion factors) were used for Zimbabwe and Yemen, because for those subprojects, expenses were reported in USD

*** Cost of vehicle hire estimated and allocated to each survey when vehicles were lent by the Minister of Health. Expenditure without cost estimation are available in S1 Dataset.

## Discussion

This study presents the costs of conducting trachoma prevalence surveys in suspected endemic populations, with support from the GTMP. It is important to have setting-specific unit costs for conducting trachoma prevalence surveys against which future surveys can be benchmarked. Indeed, the evaluation of trachoma elimination efforts requires re-estimation of TF and TT prevalence following implementation of the SAFE strategy. It is thus anticipated that the need for surveys will increase in the near future as work is ramped up to meet the 2020 trachoma elimination target. We believe that our work’s value is not limited to future efforts to plan and budget for trachoma surveys. As one of the largest infectious disease mapping exercises ever conducted, the GTMP offers important information for mapping other diseases too.

We find that the mean cost for trachoma prevalence surveys across our sample was $17,566 per EU. However, our analysis also shows that the level of expenditure for trachoma prevalence surveys was substantially different between world regions, countries, and at sub-national level (Table 5). These variations can be attributed to a number of parameters, including i) geographic location (topography, transport infrastructure, etc.); ii) demographic characteristics of the population (population density, age groups, etc.); iii) seasonal effects (dry or wet season) and iv) operational constraints (timeframe, security, logistics, unexpected events, NGOs's travel policies etc.). These parameters had an influence on the means of transportation, travel distance, per diem rates and the duration of mapping, and consequently on transport and personnel expenditure, which represented nearly 85% of the total cost of the surveys. When comparing different parts of the world, these factors were key to accounting for unit cost differences observed between the groups of subprojects in Sub-Saharan Africa, Middle-East and North Africa, and Pacific Islands and South Asia.

In the Pacific Islands and South Asia, the survey unit cost and variance (mean = US$ 24,681 per EU, standard deviation = US$ 12,777) can be explained by low density of population, security risks and relatively high price levels for commodities and services in some countries. In Vanuatu, due to the island configuration and the low density of population, a high number of clusters had to be surveyed across 17 islands in order to reach the required sample size. In addition, in both Vanuatu and Papua New Guinea, survey teams had to travel by aircraft or boat, which incurred high transport costs and increased the vulnerability of the work to weather-related delay, such as tropical storms. For security reasons, training for the Pakistan teams had to be conducted by international staff in Nigeria, increasing the cost of transportation for training participants. Finally, the high prices of commodities and services in island nations such as Solomon Islands and Vanuatu further inflated survey costs there.

In Sub-Saharan Africa, unit cost variations between sub-projects can also be explained by local conditions (mean = $16,734 per EU, SD = $9,277). The lowest survey costs were in Zanzibar, United Republic of Tanzania (US$ 7,551 per EU) and Nigeria (US$ 8,577 per EU) because of relatively high population densities and hence shorter mapping duration. On the other hand, in some regions of Ethiopia (Gambella, Beneshangul Gumuz, Ethiopia Somali and Afar), more resources were required to overcome implementation challenges caused by low population densities, unpredictable environments and harsh climatic conditions, such as extreme temperatures, volcanic activity and flooding. In Afar, for example, temperatures exceeded 45°C in the afternoon, and fieldwork was only possible for half of each day. In addition, remoteness and poor road infrastructure in some districts increased the time and resources required to reach scattered clusters. As a result, the time required to map one cluster increased substantially; resulting in high survey costs for the Gambella (US$ 40,767 per EU) and Ethiopia Somali (US$ 41,719 per EU) regions compared to other subprojects.

In contrast, with the notable exception of Darfur, Sudan, the contexts in which we worked in the Middle East and North Africa group shared quite similar characteristics, in terms of geography and population density. This underlies both the low mean unit cost ($15,252 per EU) and low variance (SD = $4,309) observed there.

There is only one previously published study on the cost of trachoma mapping: by Chen et al. [5]. Their analysis is based on data from eight national programmes in Sub-Saharan Africa, covering a total of 165 districts and 1,203 clusters. Their quoted unit costs were lower than ours, with a median cost per cluster of US$344 compared to US$625 for the GTMP, when using the same base year (2015) [12]. However, caution is advised when comparing the overall cost estimates between these two studies since the survey methodology, countries and districts mapped, and the activities or expenditure included in the analysis, all diverged; in particular, Chen et al. excluded the cost of international support in their district- and cluster-level analyses. Despite these differences, both the previous work and ours found that transport and per diems during field work were the main cost drivers for trachoma prevalence surveys. Chen et al. also found large variations in survey unit costs; ranging from an average cost of US$ 1,511 per district in Ethiopia to US$ 25,409 for Ayod in Southern Sudan. Moreover, similar local factors affecting activity costs have also been observed on a published costing study concerning lymphatic filariasis [42]. Hence, it is important for funders and program managers to recognize the significant role that local factors play when planning and budgeting.

A specific and noteworthy example of this is that the level of supervision and epidemiological support required can vary greatly from one setting to another. Supervision and coordination activities can increase the overall survey expenditure in situations where local capacity is more limited; but are likely to reduce the risk of having to remap due to poor data quality. Similarly, international experts are essential for training where local expertise is not available.

In the GTMP, the massive scale, the short timeline for implementation, as well as the drive to produce objectively gold-standard data, required a significant level of investment in global support systems including international coordination, centralised epidemiological support, and single-source provision of data management services though a small, dedicated team. The global support expenditures amounted to US$3,318 on average per district (US$ 5,668 per EU). Although a global support expenditure of US$ 5,668 per EU is substantial when compared to in-country survey expenditure, some of these costs would otherwise have been covered in-country in duplicative fashion, for example, through local costs for grant management, data collection platform set-up, epidemiologist salaries, and creation of data processing systems. Undertaking the GTMP this way would have increased its overall cost.

Moreover, the GTMP was designed to be carried out quickly so that trachoma elimination activities could start, where needed, in time to meet the global 2020 elimination target [4]. The consequence is that 1,546 districts were surveyed over three years with full GTMP support, compared to 1,115 districts mapped in 12 years prior to 2012. It is possible that survey expenditure would have been lower had survey activities been implemented only when conditions in some areas were felt to be ideal: outside the rainy season, for example, or completely separated from periods of risk for political or civil unrest. However, the higher cost of mapping to the GTMP’s tight deadline is likely to be more than offset by accelerating the elimination of trachoma[43].

Our analysis is based on rigorous financial data obtained from the GTMP for 17 countries, but had some limitations. First, the unit costs presented do not fully encapsulate the total economic cost of conducting trachoma surveys, since they do not include donations and expenditure that were not covered by the GTMP itself, particularly base salaries for health ministry employees (outside of per diems covered by GTMP), apart from vehicles which costs have been estimated and added to the appropriate survey cost (in instances when vehicles were made available by the health ministry or partners in Malawi, Nigeria, United Republic of Tanzania, Zambia and Zimbabwe). Second, budgeting and financial reporting in the GTMP was done by subproject, and it was therefore not possible to allocate expenditure items to specific district-level surveys. Unit costs presented in this paper are hence the average expenditures for EUs, districts, and clusters within a subproject. Finally, in-country survey costs were estimated based on subprojects that met our study inclusion criteria; 382 of 1,546 districts surveyed with GTMP support were excluded.

Despite these limitations, this study provides data on the setting-specific costs of conducting trachoma prevalence surveys across 52 mapping subprojects and 17 countries, and adds considerably to previous work [5]. The information we provide here can be used by endemic countries and partners to help budget for future surveys. Data on the global distribution of infectious diseases is increasingly available [44]; but significant knowledge gaps remain, even for global priorities such as malaria, HIV and tuberculosis [45–47]. We hope that our work can be used to guide studies that will start to close those gaps.

## Supporting information

S1 File List of GTMP partner organisations. (PDF)

S1 Dataset Detailed summary table of in-country expenditure per subproject. (PDF)
