# Supplementary material for: The cost of mapping trachoma: Data from the Global Trachoma Mapping Project
Source: PLoS Negl Trop Dis. 2017 Oct 18;11(10):e0006023. doi: 10.1371/journal.pntd.0006023 (PMC5675456; doi:10.1371/journal.pntd.0006023)
Supplement: S1 File — (PDF) [file pntd.0006023.s001.pdf]

Supplementary Information S1. List of GTMP Partner Organisations

Organisations:

African Medical and Research Foundation, Barraqua Institute, Blantyre Insititue for Community Ophthalmology, Brien Holden Vision Institute, The Carter Center, College of Ophthalmology & Allied Vision Sciences, Escuela Superior de Oftalmología Instituto Barraquer de América, Fred Hollows Foundation, FHI 360, Helen Keller International, International Coalition for Trachoma Control, International Trachoma Initiative (The Task Force for Global Health), Johns Hopkins University, Kilimanjaro Centre for Community Ophthalmology, Light for the World (Austria), Light for the World (Netherlands), London School of Hygiene & Tropical Medicine, Magrabi Foundation, MITOSATH, Orbis, Organisation pour la prévention de la cécité, Organizacion Panamericana de la Salud (PAHO), RTI International, Sightsavers, the World Health Organization.

National Health Ministries:

Benin, Cambodia, Chad, Colombia, Côte d'Ivoire, Democratic Republic of Congo, Egypt, Eritrea, Ethiopia, Fiji, Guinea, Kiribati, Lao People's Democratic Republic, Malawi, Mexico, Mozambique, Pakistan, Papua New Guinea, Nigeria, Republic of Congo, Senegal, Solomon Islands, Sudan, Uganda, United Republic of Tanzania, Vanuatu, Yemen, Zambia and Zimbabwe

Ethiopian Regional Health Bureaus:

Afar; Beneshangul Gumuz; Gambella; Oromia; Ethiopia Somali; Southern Nations, Nationalities, and Peoples' Region; and Tigray Regional Health Bureaus
