# Supplementary material for: The cost of mapping trachoma: Data from the Global Trachoma Mapping Project
Source: PLoS Negl Trop Dis. 2017 Oct 18;11(10):e0006023. doi: 10.1371/journal.pntd.0006023 (PMC5675456; doi:10.1371/journal.pntd.0006023)
Supplement: S1 Dataset — (PDF) [file pntd.0006023.s002.pdf]

		Survey expenditure without allocation of estimated vehicle hire cost donated and global coordination, in USD 2015			Acitvity - Proportion of Exp. (Training Included)				Category - Proportion of Mapping Exp. (Without Training Exp.)					Cost Drivers		Cost drivers in PPP	
Country	Mapping projects (n=52)	Survey Expenditure per District, in USD 2015	Survey Expenditure per EU, in USD 2015	Survey Expenditure per Cluster, in USD 2015	Training	Field Work - Mapping	Coordination - Mapping	Supervision - Mapping	Personnel - Mapping	Transportation - Mapping	Supplies - Mapping	Other - Mapping	No. of Days per Cluster	Average Vehicle Hire rate per day	Average Per diem Rate of Recorders and Graders	Average Vehicle Hire rate per day	Average Per diem Rate of Recorders and Graders
**Benin**	**Benin - Phase I**	**$8,133**	**$20,333**	**$750**	**9%**	**55%**	**34%**	**3%**	**60%**	**33%**	**5%**	**2%**	**1.2**	**$162.00**	**$55.69**	**$351.10**	**$120.69**
	Chad - Phase I	14,013	14,013	571	23%	76%	0.0%	0.3%	41%	57%	2%	0%	1.4	$202.50	$60.75	$360.72	$108.22
	Chad - Phase II	11,381	12,092	460	0%	95%	0.5%	4%	37%	57%	4%	2%	1.4	$140.54	$51.53	$270.54	$99.20
	Chad - Phase III	9,148	9,148	416	0%	81%	0.0%	19%	37%	56%	3%	3%	1.2	$129.20	$47.37	$270.54	$99.20
**Chad**	**Total**	**11,142**	**11,420**	**470**	**8%**	**84%**	**0.2%**	**8%**	**38%**	**57%**	**3%**	**2%**	**1.3**	**$157.41**	**$53.22**	**$300.60**	**$102.20**
**Egypt**	**Egypt**	**15,006**	**15,006**	**594**	**15%**	**26%**	**53%**	**7%**	**85%**	**12%**	**3%**	**0%**	**1.0**	**$77.72**	**$35.62**	**$271.29**	**$124.34**
	Ethiopia - Beneshangul Gumuz	9,997	28,564	1,031	13%	53%	24%	10%	51%	44%	4%	1%	3.2	$154.11	$30.82	$407.41	$81.48
	Ethiopia - Gambella	9,348	40,509	1,620	10%	60%	25%	5%	54%	40%	5%	1%	4.0	$141.58	$42.47	$380.25	$114.07
	**Ethiopia - Oromia Total**	**3,675**	**11,722**	**455**	**7%**	**67%**	**12%**	**14%**	**56%**	**36%**	**1%**	**6%**	**2.6**	**$141.23**	**$30.66**	**$374.81**	**$81.48**
	**Ethiopia - SNNPR Total**	**7,557**	**19,170**	**735**	**3%**	**85%**	**5%**	**7%**	**39%**	**54%**	**5%**	**2%**	**2.7**	$160.02	$30.58	$211.51	**$81.48**
	**Ethiopia - Somali Total**	**15,548**	**41,461**	**1,548**	**12%**	**69%**	**9%**	**10%**	**34%**	**54%**	**11%**	**1%**	**3.7**	**$131.75**	**$40.37**	**$373.46**	**$114.07**
	**Ethiopia - Tigray Total**	**4,607**	**12,591**	**493**	**3%**	**80%**	**7%**	**11%**	**51%**	**46%**	**2%**	**2%**	**2.0**	**$114.58**	**$34.90**	**$305.56**	**$93.02**
	**Ethiopia- Afar Total**	**12,121**	**30,855**	**1,174**	**10%**	**74%**	**6%**	**10%**	**43%**	**52%**	**4%**	**1%**	**3.6**	**$80.01**	**$41.07**	**$211.51**	**$114.07**
**Ethiopia**	**Total**	**6,538**	**19,012**	**730**	**8%**	**71%**	**11%**	**10%**	**46%**	**47%**	**5%**	**2%**	**2.8**	**$127.99**	**$35.52**	**$308.50**	**$96.99**
	Ivory Coast - Phase I	**15,960**	**21,280**	**829**	**22%**	**73%**	**3%**	**2%**	**41%**	**44%**	**15%**	**0%**	**2.4**	**$128.15**	**$84.41**	**$318.20**	**$209.58**
	Ivory Coast - Phase II	**14,214**	**14,214**	**553**	**0%**	**83%**	**1%**	**16%**	**47%**	**46%**	**7%**	**0%**	**1.2**	**$129.20**	**$84.41**	**$320.80**	**$209.58**
**Ivory Coast**	**Total**	**14,849**	**16,334**	**636**	**11%**	**78%**	**2%**	**9%**	**44%**	**45%**	**11%**	**0%**	**1.6**	**$128.67**	**$84.41**	**$319.50**	**$209.58**
	Malawi - Central, Southern	11,791	11,791	393	11%	83%	6%	0%	19%	75%	4%	3%	1.4	$215.44	$23.94	$632.10	$70.23
	Malawi - Dedza, Mulanje	11,224	11,224	468	10%	81%	2%	6%	24%	71%	3%	1%	1.7	$172.91	$28.82	$632.10	$105.35
	Malawi - Northern	9,471	11,050	368	10%	77%	9%	4%	21%	73%	4%	1%	1.4	$212.07	$23.56	$632.10	$70.23
**Malawi**	**Total**	**11,096**	**11,558**	**392**	**10%**	**80%**	**6%**	**4%**	**21%**	**73%**	**4%**	**2%**	**1.4**	**$200.14**	**$25.44**	**$632.10**	**$81.94**
	Mozambique - Nampula	11,290	16,421	684	0%	91%	0%	9%	46%	46%	4%	4%	1.5	$241.25	$80.42	$459.29	$153.10
	Mozambique - Sofala	17,402	26,103	1,105	15%	69%	10%	6%	48%	46%	6%	0%	1.7	$287.27	$79.80	$555.59	$154.33
	Mozambique - Tete province	20,590	28,826	1,201	19%	64%	11%	5%	49%	45%	2%	4%	1.8	$287.27	$79.80	$555.59	$154.33
**Mozambique**	**Total**	**16,136**	**23,369**	**978**	**12%**	**75%**	**7%**	**7%**	**48%**	**45%**	**4%**	**3%**	**1.7**	**$271.93**	**$80.00**	**$523.49**	**$153.92**
	Nigeria - Bauchi	6,052	6,052	242	15%	76%	6%	3%	51%	42%	2%	5%	1.0	$96.23	$57.74	$150.91	$90.54
	Nigeria - Benue Total	5,522	5,522	221	5%	84%	3%	8%	64%	32%	2%	2%	1.1	$53.67	$56.83	$85.51	$90.54
	Nigeria - FCT	8,738	8,738	350	19%	71%	7%	3%	55%	41%	1%	3%	1.3	$96.23	$57.74	$150.91	$90.54
	Nigeria - Gombe	6,910	6,910	277	24%	66%	7%	3%	54%	41%	1%	4%	1.0	$96.23	$57.74	$150.91	$90.54
	Nigeria - Jigawa	9,779	9,779	391	31%	58%	3%	8%	58%	36%	3%	4%	1.3	$96.23	$57.74	$150.91	$90.54
	Nigeria - Kaduna	5,340	5,340	210	15%	78%	5%	3%	49%	45%	2%	3%	1.0	$96.23	$57.74	$150.91	$90.54
	Nigeria - Kano	5,025	5,025	198	7%	86%	5%	2%	47%	40%	9%	4%	1.0	$96.23	$57.74	$150.91	$90.54
	Nigeria - Katsina	6,445	6,445	258	6%	71%	15%	7%	58%	35%	3%	4%	1.1	$96.23	$57.74	$150.91	$90.54
	Nigeria - Kogi	11,746	11,746	376	39%	59%	2%	0%	45%	43%	9%	3%	1.1	$94.72	$56.83	$150.91	$90.54
	Nigeria - Kwara	5,976	5,976	239	15%	77%	4%	3%	50%	41%	6%	3%	1.1	$94.72	$56.83	$150.91	$90.54
	Nigeria - Niger	4,971	4,971	199	12%	76%	6%	6%	52%	43%	0%	4%	1.0	$96.23	$57.74	$150.91	$90.54

	Nigeria - Kebbi and Sokoto	7,586	7,586	306	18%	76%	4%	2%	53%	45%	0%	2%	1.5	$94.72	$56.83	$150.91	$90.54
	Nigeria - Taraba	6,963	6,963	279	25%	68%	6%	2%	52%	42%	2%	4%	1.0	$96.23	$57.74	$150.91	$90.54
**Nigeria**	**Total**	**6,028**	**6,028**	**239**	**17%**	**74%**	**5%**	**4%**	**54%**	**40%**	**3%**	**3%**	**1.1**	**$96.23**	**$57.41**	**$141.56**	**$90.54**
	Pakistan - KPK and GB	11,355	12,228	459	3%	75%	14%	7%	54%	24%	13%	9%	1.3	$34.50	$17.25	$114.47	$57.24
	Pakistan - AJK	9,659	14,488	563	0%	77%	12%	11%	50%	32%	11%	8%	1.0	$59.14	$19.71	$196.24	$65.41
	Pakistan - Baluchistan and Sindh	8,715	14,526	559	10%	65%	12%	13%	58%	28%	6%	8%	1.6	$34.50	$17.25	$114.47	$57.24
	Pakistan - Punjab	8,901	8,901	330	7%	66%	17%	10%	46%	35%	5%	14%	1.3	$34.50	$13.55	$114.47	$44.97
**Pakistan**	**Total**	**9,657**	**11,267**	**423**	**5%**	**71%**	**14%**	**10%**	**52%**	**30%**	**9%**	**10%**	**1.3**	**$40.66**	**$16.94**	**$134.92**	**$56.22**
**Papua New Guinea**	**Papua New Guinea**	**43,580**	**43,580**	**1,687**	**12%**	**80%**	**6%**	**1%**	**49%**	**44%**	**4%**	**3%**	**1.0**	**$363.90**	**$58.22**	**$426.45**	**$68.23**
**Solomon Islands**	**Solomon Islands**	**13,377**	**17,835**	**653**	**26%**	**72%**	**0%**	**2%**	**42%**	**55%**	**2%**	**1%**	**1.5**	**$137.91**	**$13.79**	**$125.72**	**$12.57**
	Sudan - Khartoum	6,856	8,227	316	0%	88%	10%	2%	56%	33%	6%	5%	1.0	$156.98	$54.10	$260.83	$101.43
	Sudan - Central Darfur	11,259	15,763	788	0%	87%	11%	2%	57%	40%	3%	0%	1.4	$156.98	$122.10	$260.83	$202.87
	Sudan - East Darfur	12,106	21,186	1,059	19%	62%	16%	3%	70%	30%	0%	0%	1.5	$156.98	$122.10	$260.83	$202.87
	Sudan - North Darfur	11,586	17,378	869	0%	91%	8%	2%	62%	35%	3%	0%	1.9	$139.10	$108.19	$260.83	$202.87
	Sudan - South Darfur	13,330	15,234	773	0%	88%	10%	2%	58%	38%	4%	0%	1.4	$165.38	$108.19	$310.10	$202.87
	Sudan - West Darfur	15,035	24,056	1,114	10%	80%	7%	2.8%	61%	35%	2%	1%	1.3	$186.63	$122.10	$310.10	$202.87
**Sudan**	**Total**	**11,909**	**16,746**	**793**	**5%**	**83%**	**10%**	**2%**	**61%**	**35%**	**3%**	**1%**	**1.4**	**$160.34**	**$106.13**	**$277.26**	**$185.96**
**United Republic of Tanzania**	**Zanzibar**	**6,227**	**6,227**	**240**	**26%**	**67%**	**6%**	**2%**	**51%**	**45%**	**0%**	**4%**	**0.7**	**$73.55**	**$39.23**	**$203.02**	**$108.28**
**Vanuatu**	**Vanuatu**	**42,795**	**42,795**	**1,019**	**22%**	**58%**	**0%**	**19%**	**48%**	**45%**	**7%**	**1%**	**2.1**	**$206.28**	**$51.57**	**$175.33**	**$43.83**
	Yemen - Phase I	2,122	9,902	464	9%	70%	7%	14%	73%	25%	2%	0%	1.0	$101.71	$50.86	$268.77	$134.38
	Yemen - Phase II	3,351	11,668	486	4%	80%	6%	9%	57%	41%	2%	0%	1.7	$101.71	$50.86	$268.77	$134.38
**Yemen**	**Total**	**2,827**	**11,037**	**479**	**7%**	**75%**	**6%**	**12%**	**65%**	**33%**	**2%**	**0%**	**1.5**	**$101.71**	**$50.86**	**$268.77**	**$134.38**
**Zambia**	**Zambia**	**10,005**	**10,005**	**423**	**26%**	**56%**	**9%**	**9%**	**57%**	**33%**	**7%**	**2%**	**1.1**	**$39.20**	**$63.22**	**$116.54**	**$187.96**
	Zimbabwe - Phase I	22,255	22,255	927	10%	76%	12%	3%	41%	56%	2%	0%	2.0	$180.00	$75.00	$346.30	$144.29
	Zimbabwe - Phase II	17,795	17,795	738	4%	90%	3%	3%	37%	60%	3%	0%	2.0	$180.00	$75.00	$346.30	$144.29
**Zimbabwe**	**Total**	**20,025**	**20,025**	**832**	**7%**	**83%**	**7%**	**3%**	**39%**	**58%**	**2%**	**0%**	**2.0**	**$180.00**	**$75.00**	**$346.30**	**$144.29**
Grand Total/average		8,431	14,915	590	11%	74%	9%	7%	50%	44%	4%	3%	**1.7**	$131.87	$53.69	$276.36	$110.40
